# Effects of arbuscular mycorrhizal fungi on rice-herbivore interactions are soil-dependent

**DOI:** 10.1038/s41598-019-50354-2

**Published:** 2019-10-01

**Authors:** Lina Bernaola, Michael J. Stout

**Affiliations:** Department of Entomology, Louisiana State University Agricultural Center, Baton Rouge, 70803 Louisiana USA

**Keywords:** Symbiosis, Plant hormones, Plant physiology, Herbivory

## Abstract

The effect of soil type on establishment of arbuscular mycorrhizal (AM) fungi, and their effects on plant growth and resistance to rice pests are poorly understood. We investigated the effects of inoculation with AM fungi on rice plants in two different unsterilized field soils under greenhouse and field conditions in two consecutive years in Louisiana, United States. We tested whether inoculation with AM fungi in the two soils changed plant biomass, nutrient concentration, resistance to pests, and yields. Inoculation with a commercial formulation of AM fungi increased root colonization by fungi in all soils, regardless of soil P availability; it also increased densities of root-feeding rice water weevil larvae and growth of leaf-feeding fall armyworm larvae, but these effects were soil-dependent. Inoculation with AM fungi had no effect on N and P concentrations or rice yields. The effect on plant biomass was also soil-dependent. Our study provides evidence for the first time that inoculation with AM fungi can increase colonization of roots of rice plants, but the effects of colonization on resistance to pests and plant biomass appear to be soil dependent. Moreover, the increased susceptibility to pests of rice colonized by AM fungi does not appear to be related to nutrient concentrations.

## Introduction

Arbuscular mycorrhizal fungi (AM fungi) belong to the phylum Glomeromycota and are obligate symbionts that form mostly mutualistic associations with the roots of ca. 90% of terrestrial plants^1^. AM fungi are found in almost all soils^2,3^ and share a long history of coevolution with plants in various ecosystems, resulting in adaptation to specific geographic areas^4^. The most important function of these symbiotic associations involves the transfer of nutrients such as phosphorus (P) and nitrogen (N) by the fungus to the host plant in exchange for carbon (C), in the form of sugars and lipids^1,5^, to the fungi by the plants. Colonization by AM fungi generally promotes plant growth and also influences the interactions of plants with insect herbivores^6^, although the mechanisms remain to be elucidated. The effects of colonization by AM fungi on plant-herbivore interactions are variable; colonization by AM fungi can have beneficial, detrimental, or no effects on herbivore fitness^7–9^. For example, a detrimental effect was reported for black vine  weevil feeding on AM fungi-inoculated strawberry plants^10^, beneficial effects were reported for rice water weevil feeding on AM fungi-colonized rice^11,12^ and clover root weevil feeding on AM fungi-colonized clover plants^13^, and no effect was seen for *Junonia coenia* feeding on *Plantago lanceolata*^14^. The net effect of colonization by AM fungi on herbivores may depend on the balance of the positive effects resulting from increases in concentrations of plant nutrients and the negative effects resulting from increases in plant defenses against herbivores^13,15,16^.

Inoculation of soil with commercial AM fungi has been proposed as an alternative production practice that may contribute to more efficient nutrient use in crops^17^. Despite extensive research on the effects of AM fungi on their host plants, however, the impacts of agricultural practices that affect the soil environment, such as fertilization, tillage, and monoculture, on colonization by AM fungi are insufficiently known^4,18–20^. For instance, Barber *et al*.^21^ reported that input-intensive conventional agriculture might select for inferior fungal mutualists. Furthermore, it has been demonstrated that high concentrations of P in the soil negatively influence colonization by AM fungi in different crop plants^22^. In addition, effectiveness of soil inoculation with AM fungi varies depending on the mix of AM fungi species involved^23^. The disadvantages of soil inoculation with commercial formulations of AM fungi in agricultural fields include high application costs, the lack of positive effects of AM fungi under conditions of high nutrient (especially P) availability, and lack of effect on plant growth in some plants in some environments^24^. Despite these challenges, a meta-analysis conducted by Berruti *et al*.^23^ revealed that soil inoculation with AM fungi increased root colonization rates, and increased root colonization rates led in turn to increased root and shoot biomass, improved plant nutrition, and higher crop yields under diverse experimental conditions. Because the effects of AM fungi inoculation on plant nutrition and other plant traits vary with soil source, soil characteristics will likely influence the effects of AM fungi colonization on herbivores^6^.

Rice (*Oryza sativa* L.) is one of the world’s most important cereal crops and is also an important crop in the southern United States. In the southern U.S., including Louisiana, the majority of rice is grown under a delayed-flood cultural system in which rice is drill-seeded into dry soil, surface-irrigated as necessary to establish a stand, and flooded approximately four weeks after seeding^25^. Rice is very susceptible to different insect pests, which are one of the major problems during the growing season. The rice water weevil (*Lissorhoptrus oryzophilus*, RWW) and fall armyworm (*Spodoptera frugiperda*, FAW) are two chewing pests that can cause significant economic losses in rice production^25,26^. Current management practices to control these pests rely on the use of insecticides, but insecticides are expensive and also can cause environmental harm. Only a few studies have explored how AM fungi colonization influences the resistance of rice plants to herbivore feeding or pathogen infection and their consequences for rice fitness, with contrasting results^11,27^. Campos-Soriano *et al*.^27^ reported that inoculation with AM fungi enhanced resistance to the foliar pathogen *Magnaporthe oryzae*, while Cosme *et al*.^11^ found that females of the root-feeding RWW laid more eggs in rice plants inoculated with AM fungi, an effect that may have been caused by AM fungi-mediated increases in plant nutrient concentrations. Recently, Bernaola *et al*.^12^ demonstrated that AM fungi inoculation increases local and systemic susceptibility of rice plants to different pest organisms, including RWW and FAW, under field and greenhouse conditions. It is still not clear how soil characteristics influence colonization by AM fungi or the effects of colonization by AM fungi on the interactions between rice and its insect herbivores. In particular, whether AM fungi colonization reduces rice resistance in all soil environments is still not known.

In this study, we investigated how soil type altered the effects of inoculation of rice plants with a commercial formulation of AM fungi on plant growth and plant-herbivore interactions. We conducted field and greenhouse experiments with two soil types differing in nutrient concentration levels. A commercial formulation of AM fungi containing six species of *Glomus* was used, and effects of inoculation with AM fungi on performance of two insects were assessed.

Here, two hypotheses were tested:

(H1) The effects of inoculation with AM fungi on rice-herbivore interactions differ in soils that have different properties such as concentrations of P and/or N.

(H2) The effects of inoculation with AM fungi on plant growth, plant nutrient concentrations and yield differ in soils that have different properties.

This study represents the first study to demonstrate the soil dependency of the effects of AM fungi inoculation on plant-herbivore interactions in rice. These data will facilitate the agricultural exploitation of AM fungi-crop symbioses.

## Results

### Field experiments

#### AM fungi root colonization rates in response to AM fungi inoculation in two soil types

**Figure 1:**
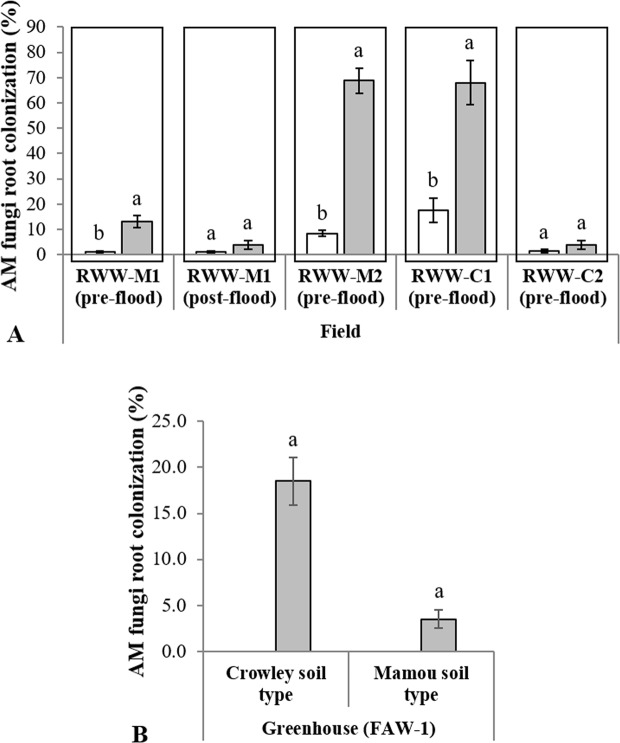
Effects of inoculation with a commercial formulation of AM fungi on percent colonization by AM fungi in rice plants grown in field (**A**) and greenhouse (**B**) conditions in two types of soil (Crowley and Mamou). Soils were either treated with mycorrhizal (*grey bars*) or with nonmycorrhizal inoculum *(white bars*). Quantification of colonization was carried out from four field experiments and one greenhouse experiment in 2014–2015. Experiments were designated as: Rice Water Weevil Mamou 1 (RWW-M1), Rice Water Weevil Mamou 2 (RWW-M2), Rice Water Weevil Crowley 1 (RWW-C1), Rice Water Weevil Crowley 2 (RWW-C2), and Fall Armyworm 1 (FAW-1). Root colonization was evaluated twice in RWW-M1 (pre- and post-flood) and once (pre-flood) in the other experiments. Percentages are means ± SE, *n* = 5. Different letters accompanying bars indicate means that differ significantly (LSD, P ≤ 0.05). See Table 1 for details of ANOVA results.

Colonization of roots of field-grown plants by AM fungi was higher in plots inoculated with commercial AM fungal inoculant than in control plots (Fig. 1A). The effect of inoculation with AM fungi was significant in RWW-M1 (29 dai, F_1,8_ = 23.04, *P* = 0.001), RWW-M2 (40 dai, F_1,8_ = 140.31, *P* < 0.0001), and RWW-C1 (44 dai, F_1,8_ = 25.57, *P* = 0.001) (Table 1). For RWW-M1, in which colonization was assessed both before and after flooding, 29-day-old rice plants inoculated with AM fungi exhibited a colonization rate of 13% before flooding. This colonization rate decreased after 13 days of flooding; colonization rates of 45-day-old (RWW-M1) rice plants inoculated with AM fungi decreased from 13 to 4% (Fig. 1A) after flooding. The largest values detected for AM fungi colonization in the field experiments were for mycorrhizal plants in RWW-C1 and RWW-M2 with 68.0% and 68.8%, respectively. Overall, our data confirmed that the inoculation with AM fungi increased the abundance of AM fungi living in rice roots grown under field conditions even in soils with different P availability.

**Table 1:** Results for the mixed models assessing effects of inoculation with AM fungi (Mycorrhizal and Nonmycorrhizal) and soil type (Crowley and Mamou) treatment on percent colonization by AM fungi, infestation by rice water weevil (RWW), root and shoot dry weights, and nutrient concentrations of rice plants grown in four experiments conducted in the field over two years (2014 and 2015).

Source of variation	RWW-M1			RWW-M2			RWW-C1			RWW-C2		
	*d.f*.	*F*	*P*	*d.f*.	*F*	*P*	*d.f*.	*F*	*P*	*d.f*.	*F*	*P*
AMF % colonization	1, 8	23.04	**0.001**	1, 8	140.3	**<.0001**	1, 8	25.57	**0.001**	1, 8	1.92	0.20
RWW density (core)	1, 16	0.92	0.35	1, 16	0.36	0.56	1, 24	11.20	**0.003**	1, 18	3.85	0.07
Shoot dry weight	1, 8	14.34	**0.02**	1, 8	1.99	0.19	1, 8	1.71	0.23	1, 6	7.73	**0.03**
Root dry weight	1, 8	9.01	**0.04**	1, 8	3.57	0.13	1, 8	6.30	**0.03**	1, 6	6.62	**0.04**
Shoot N concentration				1, 8	0.01	0.91	1, 8	0.18	0.68	1, 6	0.01	0.93
Shoot P concentration				1, 8	0.00	0.97	1, 8	14.65	**0.01**	1, 6	2.47	0.17
Root N concentration				1, 8	0.01	0.93	1, 8	2.83	0.13	1, 6	1.48	0.27
Root P concentration				1, 8	0.07	0.79	1, 8	1.40	0.27	1, 6	1.37	0.29
Adjusted yield	1, 8	0.05	0.83	1, 8	1.08	0.33	1, 8	0.00	0.96	1, 6	1.10	0.33

Experiments were designated as: Rice Water Weevil Mamou 1 (RWW-M1), Rice Water Weevil Mamou 2 (RWW-M2), Rice Water Weevil Crowley 1 (RWW-C1), and Rice Water Weevil Crowley 2 (RWW-C2).

#### Insect performance in response to AM fungi inoculation in two soil types

**Figure 2:**
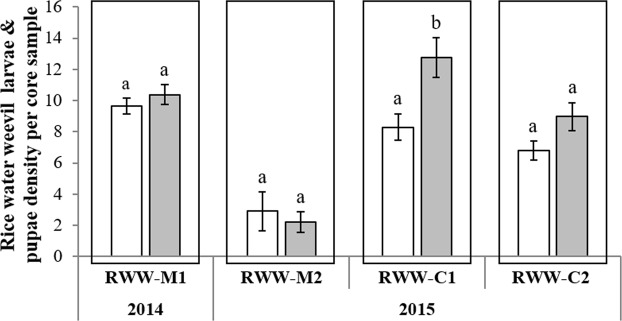
Effects of inoculation with a commercial formulation of AM fungi on the densities of rice water weevils (larvae and pupae per core sample ± SE) in rice plants grown in four field experiments of two locations with different types of soil (Crowley and Mamou) during the 2014 and 2015 growing seasons. Soils were either treated with mycorrhizal (*grey bars*) or with nonmycorrhizal inoculum (*white bars*). Experiments were designated as: Rice Water Weevil Mamou 1 (RWW-M1), Rice Water Weevil Mamou 2 (RWW-M2), Rice Water Weevil Crowley 1 (RWW-C1), and Rice Water Weevil Crowley 2 (RWW-C2). Values are means ± SE, *n* = 5. Different letters accompanying bars indicate means that differ significantly (LSD, P ≤ 0.05). See Table 1 for details of ANOVA results.

In experiments conducted at the Mamou field location (RWW-M1 & RWW-M2), densities of RWW larvae and pupae in core samples collected three and four weeks after flooding did not differ among AM fungi treatments (Fig. 2, Table 1). In the experiments conducted at the Crowley location, in contrast, larval densities were significantly higher in plots inoculated with AM fungi than in control plots in RWW-C1 (*F*_1,24_ = 11.20, *P* = 0.003). In addition, a marginally significant increase in larval densities in AM fungi-inoculated plots was observed in RWW-C2 (*F*_1,18_ = 3.85, *P* = 0.06). Increases in RWW densities in AM fungi-inoculated plots ranged from 35% in RWW-C1 to 24% in RWW-C2 (Fig. 2). Thus, the effect of inoculation with AM fungi on insect densities showed a soil dependency under field conditions.

#### Plant growth responses to AM fungi inoculation in two soil types

**Figure 3:**
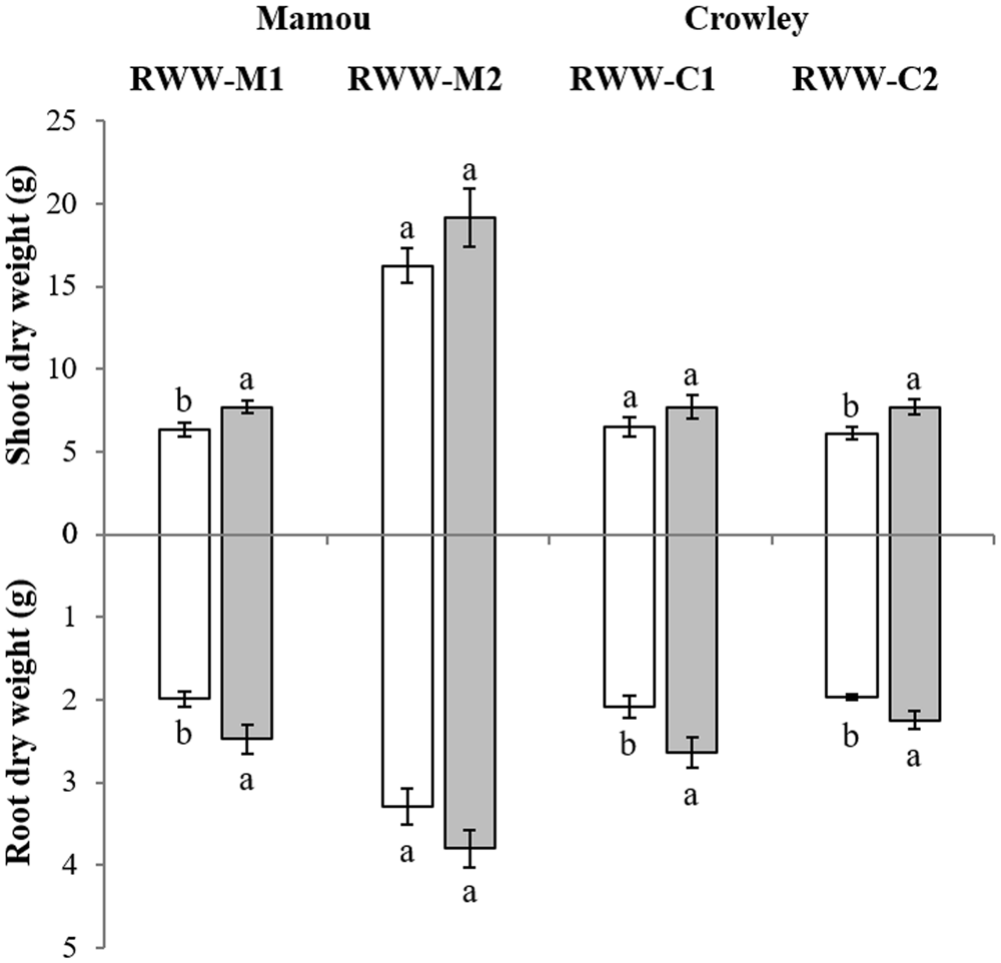
Effects of inoculation with a commercial formulation of AM fungi on shoot (above *x*-axis) and root (below *x*-axis) dry weights (grams ± S.E.) of rice plants grown in four field experiments of two locations with different types of soil (Crowley and Mamou) during the 2014 and 2015 growing seasons. Rice plants were inoculated with mycorrhizal (*grey bars*) or with nonmycorrhizal inoculum (*white bars*). Experiments were designated as: Rice Water Weevil Mamou 1 (RWW-M1), Rice Water Weevil Mamou 2 (RWW-M2), Rice Water Weevil Crowley 1 (RWW-C1), and Rice Water Weevil Crowley 2 (RWW-C2). Values are means ± SE, *n* = 5. Different letters accompanying bars indicate means that differ significantly (LSD, P ≤ 0.05). See Table 1 for details of ANOVA results.

The shoot (leaf + stem) dry weights (SDW) of plants varied with AM fungi inoculation (Fig. 3). At the Mamou location, analysis of the SDW data revealed a significant increase with AM fungi inoculation in RWW-M1 (*F*_1,8_ = 14.34; *P* = 0.02). As with SDW, root dry weights (RDW) of mycorrhizal plants were greater than that of the nonmycorrhizal plants in RWW-M1, as indicated by a significant main effect of inoculation with AM fungi (Table 1; *F*_1,8_ = 9.01; *P* = 0.04). Inoculation with AM fungi did not increase SDW or RDW in RWW-M2 (Fig. 3; Table 1), but a trend toward higher weights in mycorrhizal plants was observed. At the Crowley location, an increase in SDW (*F*_1,6_ = 6.62; *P* = 0.04) was observed in RWW-C2, but no significant effect of AM fungi inoculation on SDW was observed in RWW-C1 (*F*_1,8_ = 1.71; *P* = 0.23) (Fig. 3). A significant increase in RDW with AM fungi inoculation was observed in both experiments (RWW-C1: *F*_1,8_ = 6.30; *P* = 0.03; RWW-C2: *F*_1,6_ = 6.62; *P* = 0.04) (Fig. 3; Table 1). Overall, the highest shoot biomass increase was observed in RWW-C2 (26.0%) and RWW-C1 showed the highest increase in root biomass (27.0%).

#### Plant nutrient responses to AM fungi inoculation in two soil types

**Figure 4:**
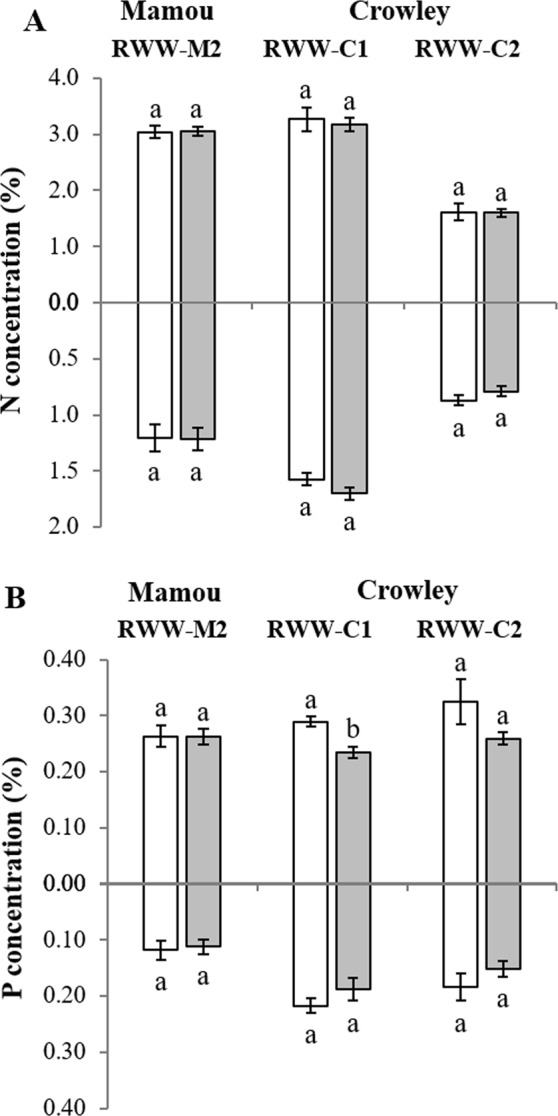
Effects of inoculation with a commercial formulation of AM fungi on concentrations of Nitrogen (**A**) and Phosphorus (**B**) in shoot (above *x*-axis) and root (below *x*-axis) of rice plants grown in three field experiments of two locations with different types of soil (Crowley and Mamou) during the 2014 and 2015 growing seasons. Rice plants were inoculated with mycorrhizal (*grey bars*) or with nonmycorrhizal inoculum (*white bars*). Experiments were designated as: Rice Water Weevil Mamou 2 (RWW-M2), Rice Water Weevil Crowley 1 (RWW-C1), and Rice Water Weevil Crowley 2 (RWW-C2). Values are mean ± SE, *n* = 5. Different letters accompanying bars indicate means that differ significantly (LSD, P ≤ 0.05). See Table 1 for details of ANOVA results.

Nutrient (N and P) concentrations in plant tissues were largely unaffected by inoculation with AM fungi (Fig. 4A,B; Table 1). The concentration of P in shoot tissues was affected by AM fungi inoculation only in RWW-C1, with a significantly higher concentration observed in the nonmycorrhizal control as compared to mycorrhizal plants (*F*_1,8_ = 14.65; *P* = 0.01; Fig. 4B).

#### Grain yield responses to AM fungi inoculation in two soil types

Grain yields were not affected by inoculation with AM fungi in any of the field experiments (see Supplementary Fig. S1 and Table 1).

### Greenhouse experiments

#### AM fungi root colonization rates in response to AM fungi inoculation in two soil types

In the greenhouse, sterilization of the soil prevented colonization by AM fungi in the roots of nonmycorrhizal plants independently of soil type in FAW-1 (Fig. 1B; root colonization was not evaluated in FAW-2). Inoculation with AM fungi significantly enhanced the percentage of root fragments colonized by AM fungi in both soil types, with inoculation leading to higher colonization in Crowley soil (19 ± 2.6%) than in Mamou soil (3.5 ± 1.0%) (Fig. 1B, Table 2). The effects of inoculation on the percentage of root colonized by AM fungi depended on soil type as shown by a highly significant ‘soil type’ x ‘AM fungi inoculation’ interaction (*F*_1, 12_ = 34.39, *P* *<* 0.0001, Table 2).

**Table 2:** Results of two-way ANOVAs assessing effects of soil source (Crowley and Mamou), inoculation treatment (Mycorrhizal and Nonmycorrhizal), and their interaction on percent colonization by AM fungi and fall armyworm (FAW) growth on rice plants grown in two experiments conducted in the greenhouse in 2014. Experiments were designated as: Fall Armyworm 1 (FAW-1) and Fall Armyworm 2 (FAW-2).

Parameter	Factor	FAW-1			FAW-2		
		*d.f*.	*F*	*P*	*d.f*.	*F*	*P*
Total % AMF Colonization	Soil type	1, 12	34.39	**<0.0001**			
	Inoculation	1, 12	73.99	**<0.0001**			
	Soil x Inoculation	1, 12	34.39	**<0.0001**			
FAW Weight gain (g)	Soil type	1, 76	15.90	**0.0002**	1, 57	16.43	**0.0002**
	Inoculation	1, 76	14.18	**0.0003**	1, 57	8.95	**0.004**
	Soil x Inoculation	1, 76	10.00	**0.002**	1, 57	0.09	0.7715

#### Effects of AM fungi inoculation on FAW growth in two soil types

**Figure 5:**
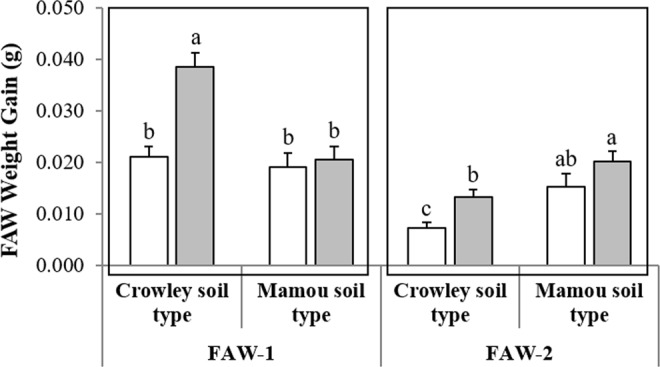
Effects of inoculation with a commercial formulation of AM fungi on weight gains of fall armyworm larvae in two greenhouse experiments using two different soil sources (Crowley and Mamou). Soils were either treated with mycorrhizal (*grey bars*) or with nonmycorrhizal inoculum (*white bars*). Experiments were designated as: Fall Armyworm 1 (FAW-1) and Fall Armyworm 2 (FAW-2). Values are means ± SE, *n* = 20. Different letters  accompanying bars indicate means that differ significantly (LSD, P ≤ 0.05). See Table 2 for details of ANOVA results.

Two-way ANOVA evaluating the effects of inoculation with AM fungi and soil type on growth of FAW larvae showed a soil dependency in effects of inoculation with AM fungi on larval growth. Weight gains of larvae were significantly affected by inoculation with AM fungi in both experiments (FAW-1: *F*_1,76_ = 14.18; *P* = 0.0003 and FAW-2: *F*_1,76_ = 8.95; *P* = 0.004) (Table 2). Weight gains of FAW larvae were also affected by ‘soil type’ in both experiments (FAW-1: *F*_1,76_ = 15.90; *P* = 0.0002 and FAW-2: *F*_1,76_ = 16.43; *P* = 0.0002) (Table 2), but the interaction of ‘soil type’ and ‘inoculation’ was significant only in FAW-1 (*F*_1,76_ = 10.00; *P* = 0.002) (Fig. 5). In both experiments, the increase in FAW growth on plants inoculated with AM fungi was seen for insects reared on plants grown in the Crowley soil but not the Mamou soil. Increases in larval growth on mycorrhizal plants in Crowley soil averaged about 46% over both experiments (FAW-1: 0.039 ± 0.003 to 0.021 ± 0.002, mean ± SE; and FAW-2: 0.013 ± 0.001 to 0.007 ± 0.001, mean ± SE) when compared to the nonmycorrhizal control plants.

## Discussion

In agricultural ecosystems, crop plants often interact simultaneously with herbivores and with AM fungi, and AM fungi and herbivores may interact indirectly through changes in their shared host plant. These tripartite interactions may be influenced by environmental factors. Building on past studies that have focused on the effects of inoculation with AM fungi on rice growth and resistance to pests^11,12^, our study investigated the effects of soil type on AM fungi-rice-herbivore interactions in two different soil types under controlled and field conditions over two years. Our results highlight the context-dependency of the effects of inoculation with AM fungi on rice growth and the interaction of rice with its herbivores.

AM fungi are known to have widespread geographical distributions^28^ and to be well-adapted to agricultural ecosystems^6^. Verbruggen *et al*.^29^ reported that compatibility with the environment is an important factor determining successful establishment of AM fungal inoculants in agricultural soils. In this study, colonization by AM fungi was successfully established using a granular commercial formulation of AM fungi over multiple years and locations. Increased root colonization levels after inoculation with AM fungi in rice fields indicated that AM fungi are compatible with different soil conditions as shown by colonization in soils with variation in pH (5.1 to 7.4), P availability (8.6 to 33.3 mg/kg), K availability (36.5 to 117.6 mg/kg), and organic matter content (0.96% to 2.25%) (Table 3), and is consistent with other studies showing that inoculation with AM fungi usually enhances root colonization by AM fungi in other plant species^17,30,31^. While these other studies focused in crop systems such as clover, alfalfa, and strawberry in different parts of the world, the results from our study support the hypothesis that inoculation with AM fungi increases root colonization in rice plants in different locations in Louisiana, and therefore perhaps, other rice-producing areas of the world as well.

**Table 3:** Properties of soils collected from two different locations for experiments conducted in 2014 and 2015.

Location	Soil name	Soil type	Location	pH	N %	P mg/kg	K mg/kg
H. Rouse Caffey Rice Research Station	Crowley	Silt loam	Acadia Parish, Louisiana	7.4 ± 0.2	0.097 ± 0.0	33.3 ± 0.5	117.6 ± 101
Kenneth LaHaye Farm	Mamou	Mowata silt loam	Evangeline Parish, Louisiana	5.1 ± 0.0	0.099 ± 0.0	8.6 ± 0.8	36.5 ± 6.5

Average values for soils collected over two years are shown (means ± SE, n = 2).

In addition to soil type, other factors may have been important in determining levels of root colonization. Since only two rice cultivars were used in the experiments, data from this study are insufficient to clearly indicate whether rice variety influenced root colonization. As seen in Fig. 1, there was no evident correlation between colonization and rice variety, but future studies should include this aspect in their experimental design, because root colonization after inoculation with AM fungi has been shown to vary among varieties within a plant species^32^. Another aspect to consider when interpreting the results of these experiments is whether colonization rates differed among the six AM fungi species in the commercial inoculum. Quantification of colonization by AM fungi in this study focused on colonization by all fungal structures, regardless of fungal species identity. Different species of AM fungi are known to vary not only in their ability to provide nutrients to plants^1^ but also in their effects on plant resistance to herbivores^33^. Irrespective of these two factors, data from this study demonstrate that AM fungi were able to influence plant biomass and yield under field experiments.

Insect performance on rice was either positively affected or not affected by inoculation with AM fungi, depending on the soil in which the plants were grown: inoculation increased densities of a root-feeding herbivore (RWW larvae) and growth of a leaf-feeding herbivore (FAW larvae) in the Crowley soil type but not the Mamou soil type. Bernaola *et al*.^12^ had previously shown that inoculation of rice plants with AM fungi increased susceptibility to RWW and FAW and a rice pathogen (sheath blight) in experiments conducted in the Crowley soil. Our results are consistent with these findings and extend them to demonstrate that this AM fungi-induced susceptibility is soil dependent. Currie *et al*.^13^ and Koricheva *et al*.^7^ have also shown root and chewing insects benefited from colonization by AM fungi, but Yang *et al*.^34^ and Gange^10^ found that colonization by AM fungi inhibited the growth of root-feeding insects. Koricheva *et al*.^7^ suggested that specialist herbivores perform better on AM fungi inoculated plants, whereas generalists do worse. However, in this study, we demonstrated that both specialist root-feeding and generalist shoot-feeding chewing insects were positively affected by AM fungi inoculation. To our knowledge, this is the first direct demonstration of soil dependence in the effect of AM fungi on rice-insect interactions. However, there are a few other studies have shown soil dependence in AM fungi-insect interactions in different crop systems^6,21^.

Increased susceptibility of rice inoculated with AM fungi to herbivores was not associated with significant effects of AM fungi on plant nutrient concentrations. In particular, inoculation with AM fungi did not affect concentrations of P or N, the nutrients most commonly studied in plant-AM fungi interactions. Similarly, Barber *et al*.^6^ found that commercial AM fungi inoculum did not change leaf nutrient content. As plant nutrient status does not explain the positive effects of AM fungi on rice-herbivore interactions in this study, changes in other plant traits such as plant defenses might have been responsible for observed effects. Future efforts could also focus on effects of colonization by AM fungi on less-studied macro- or micronutrients such as K, Na, or Zn. It has been shown that the presence of these nutrients in plant tissues can influence the performance of insect herbivores^6,35,36^.

It has been previously hypothesized that effects of AM fungi inoculation on plant growth are context-dependent. In particular, it has been found that inoculation with AM fungi increases the growth of plants under P limitation^37^, but not under conditions of P abundance. In this study, AM fungi inoculation stimulated plant growth in all field experiments and effects of plant growth were not influenced by the nutrient (N and P) status of the plant. Unlike Bernaola *et al*.^12^, who found that AM fungi inoculation increased only shoot biomass of rice plants in field and greenhouse studies, this study showed that AM fungi inoculation increased both shoot and root biomass in field experiments at the Mamou location. In general, AM fungi inoculation is known to have positive effects on plant biomass, but it is also possible that other parameters are involved, such as concentrations of other soil nutrients in agricultural fields, climatic conditions, soil microflora, P application rates, since these interactions are not fully understood yet and require future study.

Previous studies on the effect of inoculation with AM fungi inoculation on rice grain yields have been contradictory, some reporting higher yields^38–40^, lower yields, or unchanged yields as a result of inoculation with AM fungi^41^. In this study, grain yields did not differ between AM fungi treatments at either the Crowley or Mamou sites. However, the lack of an effect on grain yield may need further study, as yield components that might be affected by inoculation with AM fungi were not studied.

## Conclusions

Our study reports for the first time that effects of inoculation with AM fungi on rice growth and rice-herbivore interactions are context dependent and differ in different soil types. Future work will include identification of soil characteristics responsible for this context dependency to facilitate an understanding of how production practices mediate the potential benefits of AM fungi in rice plants. In addition, selecting more soil locations with varying properties, not only in Louisiana but also  in other rice-producing areas, will be necessary to determine the effect of inoculation with AM fungi in those areas. Understanding how inoculation with AM fungi interacts with the rice plant and how inoculation with AM fungi changes plant responses to biotic stresses is important in order to improve rice production and to promote effective and sustainable management of rice pests in ecological and agronomic contexts.

## Materials and Methods

Experiments were conducted under both field and greenhouse conditions. Field experiments were conducted at two locations with different soil properties to compare effects of inoculation with AM fungi on rice growth and RWW population densities in soils with different properties. Greenhouse experiments were conducted using soil collected from the two field locations to compare effects of inoculation with AM fungi on FAW growth rates in different soil types.

### Plants, fungi, insects, and soil sources

Two commercial varieties of rice (*Oryza sativa* L.) were used in our experiments. ‘Cocodrie’ and ‘CL111’ are both long-grain, high-yielding, early-maturing conventional varieties developed at the Louisiana State University Agricultural Center H. Rouse Caffey Rice Research Station (Crowley, Acadia parish, LA, USA). ‘Cocodrie’ is susceptible to RWW and grown widely in the southern U.S., and was chosen for this study because it had been used in previous studies of rice-mycorrhizal-herbivore interactions^12^. ‘CL111’ is an herbicide-tolerant variety chosen because it was the most widely grown rice variety in Louisiana in 2014–2015. Seeds of rice were kindly provided by the breeding and foundation seed program at the LSU AgCenter H. Rouse Caffey Rice Research Station.

A commercial inoculum of AM fungi containing only AM fungal propagules (ECOVAM^TM^ VAM Endo Granular, Horticultural Alliance Inc., Sarasota, FL, USA) was selected to establish and promote symbiosis with rice plants in both field and greenhouse experiments. The inoculum consisted of spores, hyphae and colonized root fragments of six species of AM fungi as described in Bernaola *et al*.^12^. The six AM fungi species (*Rhizophagus irregularis, Funneliformis mosseae, Glomus deserticola, Rhizophagus fasciculatum, Sclerocystis dussii, and Glomus microaggregatum*) were originally obtained from the International Culture Collection of (Vesicular) Arbuscular Mycorrhizal Fungi (INVAM, West Virginia University, USA). The AM fungi propagules were carried in inert material consisting of a uniform mixture of zeolite, pumice, vermiculite, perlite and attapulgite. The formulated material contained an average of 132 spores of AM fungi (all six species) per gram, in addition to hyphae and colonized root fragments.

The rice water weevil (RWW; *Lissorhoptrus oryzophilus* Kuschel; Coleoptera: Curculionidae) is the most destructive insect pest of rice in the United States^25,42,43^. Field experiments relied on natural infestations of RWWs, which are abundant at the field sites^25^. Adult RWWs feed on young rice leaves, producing longitudinal scars, and females lay eggs primarily in leaf sheaths of flooded rice plants. Larval RWW have a strong impact on rice yields by feeding on roots of flooded rice^11^.

Larvae of the fall armyworm (FAW, *Spodoptera frugiperda* J.E. Smith; Lepidoptera: Noctuidae) were obtained from a colony maintained continuously on meridic diet in a laboratory. The colony originated from larvae collected in rice fields near Crowley, LA, in 2013. Adult female armyworms oviposit eggs on leaf blades and other substrates, giving rise to larvae that feed on leaves^26^. The diet used for rearing of larvae was Fall Armyworm Diet (Southland Products Incorporated, Lake Village, AR, USA). The colony was maintained under controlled environmental conditions (L14: D10, 28 ± 2 °C, 38 ± 2% R.H).

Field experiments were conducted at, and soils were sourced from, two locations in southwest Louisiana. The first location was the LSU AgCenter H. Rouse Caffey Rice Research Station (Crowley, Acadia Parish, 30°14′22″N, 92°20′46″W), while the second location was in a farmer’s field in Mamou, Louisiana (Evangeline Parish, 30°38′28″N, 92°27′33″W). The physicochemical properties of soils from the two sites were analyzed by the LSU AgCenter Soil Testing & Plant Analysis Laboratory (STPAL, LSU, Baton Rouge, LA). The soils varied in their properties as shown in Table 3. Notably, soil P and K were at least four and three times higher in the Crowley soil than in the Mamou soil, respectively. The Mamou soil was more acidic (pH 5.1) than the Crowley soil (pH 7.4).

For greenhouse experiments, soils were collected from the top 6 inches of topsoil at each of the field sites described, in early summer in 2014. Before used in greenhouse experiments, soil was sterilized at 121 °C for 60 min. After sterilization, Crowley and Mamou soils had a pH of 7.7 and 4.7, a total P content of 31.5 and 10.9 mg/kg, and a total K content of 132.4 and 44.5 mg/kg, respectively.

### Field experiments

Previous small-plot experiments conducted at the Crowley location established that inoculation with a commercial formulation of AM fungi often increased the susceptibility of rice to RWW^12^. For the current study, four small-plot field experiments (one in 2014 and three in 2015) were carried out to evaluate the effects of soil type on the susceptibility of RWW to AM fungi inoculation. Experiments were designated as: Rice Water Weevil Mamou 1 (RWW-M1), Rice Water Weevil Mamou 2 (RWW-M2), Rice Water Weevil Crowley 1 (RWW-C1) and Rice Water Weevil Crowley 2 (RWW-C2) (Table 4).

**Table 4:** Planting and insect sampling dates for field and greenhouse experiments conducted over the 2014 and 2015 growing seasons to evaluate the effects of inoculation with AM fungi on the performance of rice water weevil and the growth of fall armyworm on rice plants. Experiments were designated as: Rice Water Weevil Mamou 1 (RWW-M1), Rice Water Weevil Mamou 2 (RWW-M2), Rice Water Weevil Crowley 1 (RWW-C1), Rice Water Weevil Crowley 2 (RWW-C2), Fall Armyworm 1 (FAW-1), and Fall Armyworm 2 (FAW-2).

Year	Experiment	Planting date	Flooding date	AM fungi sampling date	RWW core sampling dates
*Field*					
2014	RWW-M1	21^st^ April	23^rd^ May	20^th^ May & 6^th^ June	12^th^ & 18^th^ June
2015	RWW-M2	31^st^ March	15^th^ May	5^th^ May	5^th^ & 9^th^ June
	RWW-C1	25^th^ March	15^th^ May	8^th^ May	9^th^, 16^th^ & 23^rd^ June
	RWW-C2	4^th^ May	10^th^ June	5^th^ June	30^th^ June, 6^th^ & 13^th^ July
**Year**	**Experiment**	**Planting date**	**AM fungi sampling date**	**FAW final weight measurements**	
*Greenhouse*					
2014	FAW-1	1^st^ Jul	30^th^ July	11^th^ August	
	FAW-2	26^th^ Aug	—	17^th^ October	

All experiments were laid out in a completely randomized design (CRD) and each experiment included two treatments, one in which plots were inoculated with AM fungi and one in which plots were inoculated with a nonmycorrhizal control. Each of the two treatments was replicated five times, resulting in 10 plots per experiment. For the nonmycorrhizal control, plots were seeded into soils treated with a mock inoculum, which contains all the inert ingredients of the AM fungi inoculum but without the fungi. For the mycorrhizal treatment, rice seeds were sown in soil inoculated with live AM fungi. Mock or live inoculum was applied to the surface of the soil after planting and gently raked in to incorporate the live or mock inoculum into the upper 2.5 cm of the soil. Because rice was grown in the field, soil was not sterilized and likely contained native AM fungi.

Rice was drill-seeded on the dates specified in Table 4 at a rate of 85 g (68 kg/ha) of seeds per plot. Plots measured 1.4 m × 4.9 m. Each plot was inoculated with 17 kg of mock inoculum or live inoculum. The inoculum amounts used in both years corresponded to approximately 2.2 million AM fungi spores per plot. Plots were flushed with well water as necessary for the first month after seeding to establish stands of rice. After allowing the plants to grow without a flood for approximately one month, permanent floods were applied on the dates specified in Table 4. Plants possessed 4–5 leaves (early tillering) at permanent flooding.

Densities of RWW larvae and pupae were determined after permanent flooding by taking root/soil core samples from each plot^44^. The core sampler was a metal cylinder with a diameter of 9.2 cm and a depth of 7.6 cm attached to a metal handle. Core sampling was conducted twice at the Mamou site and three times at the Crowley site for all experiments. All core sampling was conducted between three and five weeks after permanent flood. Dates of core samplings are shown in Table 4. For each core sampling, two or three (2014) and three or four (2015) core samples were taken from each plot. Core samples were transported in plastic bags to a processing facility, where each sample was placed into a 40-mesh screen sieve bucket to wash the soil and larvae from roots. Buckets with rinsed samples were placed into basins of salt water, and larvae and pupae were counted as they floated to the water surface^45^. RWW counts from two to four core samples from each plot per sampling date were averaged to obtain mean densities of immature weevils (larvae and pupae) per core sample.

### Greenhouse experiments

Additional experiments were conducted in the greenhouse to further test the hypothesis that differential effects of inoculation with AM fungi on susceptibility to insects were attributable to differences in the properties of soil at the two field sites. Two laboratory feeding assays were conducted in 2014 using cut leaf material to determine whether mycorrhizal inoculation affected growth of FAW larvae. Experiments were designated as Fall Armyworm 1 (FAW-1) and Fall Armyworm 2 (FAW-2) (see Table 4). ‘Cocodrie’ rice plants were grown under two treatments, namely mycorrhizal and nonmycorrhizal.

All plants were grown in 2 liter round (15 cm diameter) plastic pots (Hummert International, Earth City, MO) filled with sterilized soil from one of the two field sites to which 50 g of mycorrhizal inoculum or 50 g mock inoculum were added. The inoculum was thoroughly mixed with the soil before filling pots. Four rice seeds were sown per pot and a total of 25 pots per treatment were set up. Plants were maintained under greenhouse conditions with temperatures ranging from 25 °C to 35 °C and ambient lighting. Rice seedlings were thinned to two plants per pot two weeks after planting. Leaves for FAW feeding assays were taken from plants that were three weeks old; plants possessed three or four leaves at the time experiments were initiated. Because these experiments were conducted with rice at an early stage of growth, additional fertilizer was not necessary for satisfactory plant growth.

Neonate FAW that had eclosed within 24 hours were used for feeding assays. Feeding assays were conducted in 9 cm plastic petri dishes lined with moistened cotton batting to maintain turgor in excised tissues. Youngest fully-expanded leaves were removed from plants of each treatment group using scissors, transported on ice to the laboratory, cut into ca. 7 cm pieces, and placed in petri dishes. Three neonates were placed together in each petri dish with foliage and allowed to feed on excised leaf material for 10 days in each experiment. Larvae were observed daily to ensure they were not food-limited and leaves were changed every other day (every day for larvae in later stages). After ending the feeding assay, larvae were starved for three hours to ensure that the larval gut was emptied before final masses were determined. The mean mass of the remaining larvae in each petri dish was calculated. Weight gain (final weight) was recorded as the response variable and initial weight of neonates was considered to be zero. For each experiment, 20 petri dishes (replicates) were used for each treatment for a total of 80 observations for each of the FAW experiments. Insects that died during feeding assays were excluded.

### Quantification of mycorrhizal colonization

In order to verify the effectiveness of AM fungi inoculations, the extent of AM fungi colonization was measured in each experiment. Root colonization by AM fungi was evaluated twice during plant development in RWW-M1, before and after flood establishment. Root colonization was evaluated once (before flooding) in the other field (RWW-M2, RWW-C1 and RWW-C2) and greenhouse (FAW-1) experiments. Sampling was conducted by taking 9.2 cm diameter soil-root cores from field plots, or washing the roots from greenhouse pots containing entire rice plants. For the purpose of this study, one soil-root core (field experiments) or pot (greenhouse experiments) represented one plant sample. Ten root samples from each experiment were randomly collected from five plots or pots of each treatment group per sampling date (Table 4). Each soil-root core or pot, containing two to four plants, was placed in plastic bags (one core per bag) and taken to the laboratory to be processed for root staining.

The trypan blue method of Koske and Gemma^46^ was used with minor modifications for root staining of AM fungi colonization. Clearing and staining procedures require root samples to be washed from soil to remove all soil particles and then separating root and shoot tissues. For subsampling, roots from each soil-root core or pot were cut into 2-cm-long segments and placed in tissue processing cassettes (Ted Pella, Redding, CA). At least 250 small root pieces per root sample (either soil-root core or pot) were cleared in 10% KOH in a water bath at 90 °C for 20 min. Clear pieces of roots were rinsed five times with tap water to remove KOH, and roots were immersed in 2% HCl at room temperature for 10–15 min to ensure the roots were effectively acidified for staining. Cassettes containing roots were immediately stained with 0.05% trypan blue (Sigma-Aldrich, St. Louis, MO, USA) by incubation overnight and then transferred to vials containing lactoglycerol at 4 °C to allow excess stain to leach out of the roots. Stained root samples were stored in destaining lactoglycerol solution for 48 h before being mounted in the same solution on a microscope slide.

**Figure 6:**
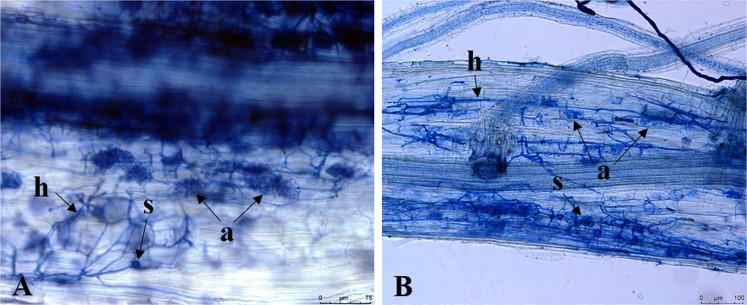
Root fragments stained with trypan blue showing arbuscular mycorrhizal fungi structures in rice plants. Light micrographs of mycorrhizal inoculated-root fragments from some experiments conducted in 2015 show: (**A**) Hyphae (h), arbuscule (a), and spore (s). (**B**) Hyphae, arbuscule, and spore (s).

The method of McGonigle *et al*.^47^ was used with modification for quantifying the abundance of AM fungi colonization. Five microscope slides for each root sample, each containing ten 2-cm-long root fragments, were mounted after staining on microscopic slides. Root fragments were randomly selected from each root sample and are representative of the whole root system as it was not possible to separate root types. A total of 50 root samples were collected from four field experiments and 20 root samples from one greenhouse experiment. For each root sample, 50 stained root fragments (250 stained root fragments per treatment) were examined with a compound microscope (Olympus CH2, Tokyo, Japan) at 40X magnification in order to confirm the levels of AM fungi colonization. The presence of blue-stained mycorrhizal structures in the root fragments including intraradical aseptate hyphae linked to either arbuscules or vesicles/spores were scored as colonized by AM fungi^48^ (Fig. 6). Photos of AM fungi structures on mycorrhizal colonized roots were taken using a microscope-mounted 5.0-megapixel digital camera (Leica DFC480, Cambridge, UK). Percent of root fragments with AM fungi colonization was averaged per treatment for the analyzed experiments.

### Effects of AM fungi on rice growth and nutrient concentrations

To determine the effect of inoculation with AM fungi on plant biomass, entire plants were collected from AM fungi-inoculated and control plots. Four to five weeks after planting, entire plants were harvested from field plots by taking one soil-root core per plot. Entire plants were also collected from pots in greenhouse experiments (see above). Soil was washed from roots, and the shoot (leaf + stem), and root portions of plants were separated and blotted dry with a paper towel. Plant material was dried in an oven to constant weight (60 °C for 1 week) and shoot dry weight (SDW) and root dry weight (RDW) were measured for each plant.

To evaluate whether AM fungi inoculation affected nutrient concentrations in leaves and roots of rice plants, the same plant tissue samples collected for plant biomass were used for plant analysis. After the samples were dried and weighed, portions of plants were submitted to the LSU AgCenter Soil Testing & Plant Analysis Laboratory (STPAL, LSU, Baton Rouge, LA) to determine nutrient concentrations in shoot and root tissues. N and C content were determined by dry combustion using a LECO TruSpec^TM^ CN analyzer (LECO Corp., St. Joseph, MI, USA), while concentrations of the remaining nutrients (Ca, Mg, S, P, K, Al, B, Cu, Fe, Mn, Na and Zn) were determined by inductively coupled plasma (ICP) analysis.

To assess the effect of the AM fungi inoculation on plant growth (field experiments only), mycorrhizal growth responses (MGR) were calculated as effect sizes using the individual biomass dry weights of the AM fungi-inoculated plants and mean biomass dry weight values of mock-inoculated control plants (average of five plots per treatment).

$$ \% {MGR}=\frac{{Dry}\,{weight}({\rm{AM}}\,{\rm{fungi}}-{\rm{inoculated}})-{mean}\,{dry}\,{weight}({\rm{mock}}-{\rm{inoculated}})}{{mean}\,{dry}\,{weight}({\rm{mock}}-{\rm{inoculated}})}\times 100$$

Yield data were obtained only for field experiments. Four rice rows in the center of each plot were harvested at grain maturity by a mechanical combine and grain yield (expressed at 12% moisture) was calculated.

### Statistical analyses

Prior to analysis, data were analyzed to verify that they met assumptions of normality. Statistical analyses were conducted using SAS 9.4 (SAS Institute 2014). For field experiments, the effect of AM fungi inoculation on root colonization rates, RWW larval densities, plant biomass, nutrient concentrations, and grain yields were analyzed separately with analysis of variance (ANOVA) in PROC MIXED^49^. Data for RWW larval densities were analyzed independently each year by repeated measures ANOVA. Inoculation treatment was used as fixed effect and block as a random effect.

For greenhouse experiments, the effect of AM fungi inoculation on root colonization rates and FAW weight gain were analyzed by two-way ANOVAs with ‘soil type’ (Crowley and Mamou), ‘Inoculation treatment’, and their interaction as fixed effects, with replication as a random effect. Means were separated using the least significant difference (LSD, P ≤ 0.05) test.

## Supplementary information

Figure S1

## Data Availability

The datasets generated and/or analyzed during the current study are available from the corresponding author on request.
