# Supplementary material for: Effects of arbuscular mycorrhizal fungi on rice-herbivore interactions are soil-dependent
Source: Sci Rep. 2019 Oct 1;9:14037. doi: 10.1038/s41598-019-50354-2 (PMC6773947; doi:10.1038/s41598-019-50354-2)

Effects of arbuscular mycorrhizal fungi on rice-herbivore interactions are soil-dependent

Lina Bernaola and Michael Stout

Supporting Information

Supplementary Figure S1. Influence of arbuscular mycorrhizal fungi inoculation on yields from field experiments in two different locations during 2014-2015. Yields were adjusted to 12% moisture. Rice plants were inoculated with AMF (*grey bars*) or with NM control inoculum (*white bars*). Bars represent means of five ± SE. Bars and upper case letters at the column head indicate that means differ significantly (LSD, $P \leq 0.05$).

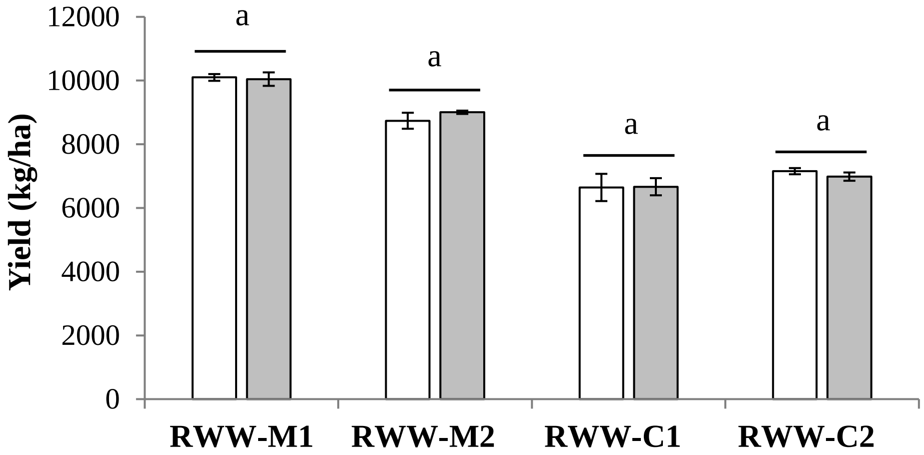

Supplement: Supplementary file 1 — Figure S1 [file 41598_2019_50354_MOESM1_ESM.pdf]
